# Transcriptome analysis of *Listeria monocytogenes* exposed to biocide stress reveals a multi-system response involving cell wall synthesis, sugar uptake, and motility

**DOI:** 10.3389/fmicb.2014.00068

**Published:** 2014-02-28

**Authors:** Aidan Casey, Edward M. Fox, Stephan Schmitz-Esser, Aidan Coffey, Olivia McAuliffe, Kieran Jordan

**Affiliations:** ^1^Teagasc Food Research CentreFermoy, Ireland; ^2^CSIRO Animal Food and Health SciencesWerribee, VIC, Australia; ^3^Department of Farm Animals and Veterinary Public Health, Institute of Milk Hygiene, University of Veterinary MedicineVienna, Austria; ^4^Department of Biological Sciences, Cork Institute of TechnologyCork, Ireland

**Keywords:** *Listeria monocytogenes*, RNA-Seq, benzethonium chloride, transcriptome, gene expression, biocide stress

## Abstract

*Listeria monocytogenes* is a virulent food-borne pathogen most often associated with the consumption of “ready-to-eat” foods. The organism is a common contaminant of food processing plants where it may persist for extended periods of time. A commonly used approach for the control of *Listeria monocytogenes* in the processing environment is the application of biocides such as quaternary ammonium compounds. In this study, the transcriptomic response of a persistent strain of *L*. *monocytogenes* (strain 6179) on exposure to a sub-lethal concentration of the quaternary ammonium compound benzethonium chloride (BZT) was assessed. Using RNA-Seq, gene expression levels were quantified by sequencing the transcriptome of *L. monocytogenes* 6179 in the presence (4 ppm) and absence of BZT, and mapping each data set to the sequenced genome of strain 6179. Hundreds of differentially expressed genes were identified, and subsequent analysis suggested that many biological processes such as peptidoglycan biosynthesis, bacterial chemotaxis and motility, and carbohydrate uptake, were involved in the response of *L. monocyotogenes* to the presence of BZT. The information generated in this study further contributes to our understanding of the response of bacteria to environmental stress. In addition, this study demonstrates the importance of using the bacterium's own genome as a reference when analysing RNA-Seq data.

## Introduction

*Listeria monocytogenes* is a virulent food-borne pathogen that is responsible for the bacterial infection listeriosis. Listeriosis is a relatively rare disease, having an incidence of between 2–10 reported cases per million people every year in Europe (Holck and Berg, 2009), and approximately 2000 hospitalizations per annum in the United States (Guenther et al., 2009). However, it has a significantly high mortality rate of 20–30% (Vázquez-Boland et al., 2001), making it one of the most devastating food-borne bacterial pathogens.

The main vehicle for transmission of *L. monocytogenes* to the human host is through the consumption of contaminated food products. *L. monocytogenes* is considerably more resilient than many other bacteria associated with food, being capable of multiplying at low temperatures, low pH and high salt concentration (Gandhi and Chikindas, 2007). These characteristics give the organism a competitive advantage in certain types of foods, particularly chilled foods that are highly processed and have a long shelf life. Due to its ubiquitous nature, *L. monocytogenes* is a common contaminant of food processing facilities. The organism has proven quite difficult to eradicate, and several subtypes of the bacterium are able to persistently colonize food-processing environments over extended periods of time (Fox et al., 2011a,b). This observation of persistence has very serious consequences for food safety considering that strains which can successfully persist in such environments could and often can contribute to an increased risk of cross-contamination of products. The downstream consequences of this include financial losses due to mass product recall and indeed the possibility of human infection and disease outbreak, following consumption of contaminated products (Laksanalamai et al., 2012). An in-depth study of persistent strains of *L. monocytogenes* is however quite difficult to achieve, considering that the only criterion for defining a strain as persistent is through its re-isolation from a food processing environment on numerous occasions over a prolonged period (Kastbjerg and Gram, 2009).

Control of *L. monocytogenes* in the food processing environment is of paramount importance to industry if the human and economic consequences of a *L. monocytogenes* outbreak are to be minimized. A common method for the control and removal of pathogenic organisms from the processing environment is through the application of quaternary ammonium compounds (QAC), which are non-corrosive, cationic agents, used frequently and in high concentrations as biocides. A study on the minimum inhibitory concentrations (MICs) of a QAC required to prevent growth of *L. monocytogenes* (Lundén et al., 2003), indicated that a QAC concentration of between 0.63 to 5.0 μg/ml was sufficient to prevent the bacterium from proliferating. In industry, it is commonplace to find dilutions of about 1000 mg/L being used when applying QACs to machinery for disinfection (Meyer, 2006). While, in theory, the high concentration of QAC ensures complete eradication of any pathogenic bacteria from the surface of industrial equipment, *L. monocytogenes* has been shown to survive and adapt when exposed to sub-lethal concentrations of these disinfectants. A recent study investigated the transcriptional response of two different strains of *L. monocytogenes* (namely a persister isolated from cheese production environment and a non-persister isolated from cheese) on exposure to sub-lethal concentrations of the QAC, benzethonium chloride (BZT). Using a closely related genome as a reference for the study (*L. monocytogenes* strain F6854), Fox et al identified numerous genes which exhibited a marked increase in expression levels on BZT exposure, including those involved in the cell wall reinforcement, sugar metabolism, transcription, pH regulation and biosynthesis of cofactors (Fox et al., 2011a,b).

The aim of this study was to assess the global response of a persistent strain of *L. monocytogenes* on exposure to sub-lethal concentrations of BZT using transcriptome sequencing and subsequent RNA-Seq analysis. Gene expression levels of strain 6179 were compared in the presence or absence of BZT using the 6179 genome sequence as the reference genome.

## Materials and methods

### mRNA enrichment from *listeria monocytogenes*

A persistent *L. monocytogenes* isolate from farmhouse cheese, strain 6179, was grown statically at 14°C in tryptic soy broth (TSB) to early stationary phase, under two separate experimental conditions; in the presence (4 ppm) and absence (0 ppm) of BZT (Sigma Aldrich, Co. Wicklow, Ireland). BZT was prepared by dissolving in TSB, filter-sterilizing the solution through a 0.45 μm filter (Sarstedt, Co. Wexford, Ireland), and adding this solution to yield a final concentration of 4 ppm BZT in the test sample. RNA was isolated using the Qiagen RNeasy midi kit (Qiagen, Crawley, West Sussex, UK) with some modifications. Basically, to each 50 ml sample (one test and one control), two volumes of RNAprotect (Qiagen) were added. Samples were centrifuged for 20 min at 3074 × g and the resultant pellets resuspended in 500 μl TE buffer containing 5 mg ml^−1^ lysozyme (Sigma Aldrich). Samples were loaded onto the Qiagen RNeasy columns and centrifuged for 5 min at 1902 × g. The resultant flow-through was applied to the column a second time to maximize RNA yield. For elution, 150 μl of nuclease free water (MyBio, Kilkenny, Ireland) was added to each column, which were allowed to stand for 5 min at room temperature, before being centrifuged at 1902 × g for 5 min. The eluate was reapplied to each column, columns were allowed to stand for a further 5 min at room temperature, before once again being centrifuged at 1902 × g for 5 min. RNA purity was assessed using the Thermo Scientific NanoDrop, while RNA integrity was checked by electrophoresis. mRNA enrichment was carried out using the Ambion MicrobExpress kit (Bio-Sciences, Co. Dublin, Ireland) with the following modifications. RNA precipitations were performed using 3 volumes of 100% ethanol and 0.1 volumes of 3 M sodium acetate, followed by incubation at −70°C for 30 min. RNA was recovered following centrifugation at a temperature of 4°C, and at a speed of 18,400 × g for 45 min. Resuspension of RNA following precipitation was done in 15 μl TE buffer. For the RNA-annealing step, 10 μg of RNA was used, with oligonucleotide capture being performed at 37°C for 30 min. Enriched mRNA was ultimately resuspended in 25 μl of nuclease-free water.

### Transcriptome sequencing

Enriched mRNA samples for each of the two experimental growth conditions were sent for commercial sequencing on the Illumina sequencing platform (Source Bioscience UK Ltd, Nottingham, UK). The raw sequence data generated, containing the sequenced mRNA reads, was filtered to discard any reads that did not meet overall quality values as determined by the Illumina chastity filter (Source Bioscience UK Ltd). Output data generated from sequencing was stored in the standard FASTQ format for use as input for subsequent analysis.

### RNA-Seq analysis of sequenced data

RNA-Seq analysis of sequence data generated was undertaken using the Arraystar QSeq application of the DNAStar Lasergene Genomics Suite (DNASTAR, Inc., Madison, USA). Experimental inputs for the analysis included the previously described FASTQ files containing sequenced reads for each experimental condition, and two Genbank files which contained reference genome and reference plasmid features for the complete sequence of strain 6179. Sequenced reads generated for each of the two experimental conditions were mapped against the reference genome of strain 6179 using the QSeq application in order to quantify gene expression levels of the bacterium for each experimental condition. Mapped read count normalization was applied to the data based on the number of reads per kilobase of coding sequence per million mapped reads (RPKM) (Mortazavi et al., 2008).

## Results and discussion

*L. monocytogenes* strain 6179 is a persistent strain of the 1/2a serotype originally isolated from a cheese processing environment. It is classed as a persistent strain due to the frequency at which it has been isolated from the same environment (greater than 50% of sampling dates over a 2-year sampling period, and on multiple occasions over a period of 12 years)(Fox et al., 2011a,b). Considering the fact that strain 6179 is of the 1/2a serotype, and is one of three serotypes of the bacteria (along with 1/2b and 4b) found to be prevalent in human foodborne illness (Kathariou, 2002), understanding the genetic basis by which strains such as 6179 are capable of surviving detergent treatment is of high value to the ready-to-eat food processing industry, given the threat it poses for product contamination. The aim of this study was to elucidate the molecular response of a *L. monocytogenes* strain with increased resistance to QACs on exposure to sub-lethal BZT. This knowledge will provide insights into potential mechanisms that may be used by *L. monocytogenes* to achieve persistence in the food processing environment, and to ultimately contribute to how *L. monocytogenes* contamination will be addressed in the future.

This study quantified gene expression levels in strain 6179 following growth of the bacterium in the presence or absence of sub-lethal concentrations of BZT, and subsequently compared the differences in expression levels between the two states, in order to elucidate how the bacterium responds and adapts to the stress of exposure to this particular industrial detergent. In total, the analysis identified approximately 600 genes which demonstrated a 4-fold or greater change in relative expression in the BZT-treated sample compared to the control. Differential regulation of a factor of 7.5-fold or greater was observed in the case of over 141 genes (a similar figure to the ca. 100 genes reported above this threshold in the previous study by Fox et al.). The genes which exhibited the greatest change in relative expression between experimental states function in a range of different cellular processes.

Table 1 illustrates a comparative analysis between the results reported in this particular study and those reported in a similar study by Fox et al. (2011a,b). The difference in approach between this study and that of Fox et al. (2011a,b) concerned both the reference strain against which the mRNA reads were mapped, and indeed the type of software used to carry out the RNA-Seq analysis. While the study by Fox et al. (2011a,b) used a closely related genome as a reference (*L. monocytogenes* strain f6854), the genome of 6179 has since been fully sequenced, and was available for use as a reference genome in this study. In using strain F6854 as a reference genome, the previous study was quite valuable in identifying some of the key responses utilized by strain 6179 in order to tolerate exposure to BZT. However, it was limited in that response mechanisms that are specific to strain 6179 (or indeed mechanisms that are only shared by strain 6179 and certain other strains) would not have been detected by the RNA-Seq analysis if the reference strain chosen did not share them. Additionally, the authors believe that a greater degree of confidence in the precision and accuracy of the resulting read counts is warranted when using 6179's own genome as a reference. Therefore, certain genetic responses which did not meet the criteria for reporting in the previous study are recognized as important and worthy of reporting in this study. In this sense, using 6179's own fully sequenced genome as a reference provided a more comprehensive picture of how the bacterium responds to BZT exposure, and this is reflected in the results obtained in this study. Both of the aforementioned reference strains are persistent isolates of the same 1/2a serotype, and have a relatively similar genome size of 2.9 Mb and 3.0 Mb respectively. However, strain 6179 was isolated from a cheese production facility and contains a large 60 kb plasmid, while strain F6854 was first isolated from a turkey frankfurter processing plant, but does not contain a plasmid (Nelson et al., 2004).

**Table 1:** **Functional Groups containing genes that were reported to be considerably up-regulated upon exposure of the bacterium to BZT: comparison of this study with that of Fox et al. (2011a,b)**.

**Functional group**	**Considerable up-regulation of genes in:**	
	**This study (6179 v 6179)**	Fox et al., 2011a,b; (6179 v f6854)
Peptidoglycan biosynthesis	+	+
Chemotaxis	+	
Flagellar assembly	+	
Fatty acid metabolism/biosynthesis	+	+
Phosphotransferase system	+	+
Cobalamin biosynthesis	+	

The functional groups of genes for which there was a substantial difference in expression compared to controls are indicated for each of the studies (Table 1). While a considerable up-regulation in genes involved in peptidoglycan biosynthesis, fatty acid metabolism and the phosphotransferase system were observed in both studies, the marked increase in chemotaxis, flagellar assembly and cobalamin biosynthesis genes is only reported for this study, where mRNA reads for strain 6179 were mapped back to the bacterium's own fully sequenced genome. Relative expression levels of genes on the large 60 kb plasmid were also assessed, with the only noteworthy changes including an up-regulation of the *repB* gene by 4.4-fold, and an up-regulation of the cadmium-transporting ATPase *cadA* by 2.45-fold compared to controls.

### Chemotaxis and flagellar assembly

A number of genes involved in the signaling cascade of bacterial chemotaxis were considerably up-regulated in expression when strain 6179 was grown in the presence of BZT. Among these, a gene coding for the methyl-accepting chemotaxis protein (MCP), a transmembrane protein involved in detection and transduction of extracellular sensory signals (Derr et al., 2006), showed increased expression of greater than 6-fold compared to the control. Likewise, the two-component system, *cheA*/*cheY*, which is involved in extracellular signal transduction through the bacterial cell (Dons et al., 2004), was also up-regulated, by factors of approximately 4-fold and 7-fold respectively. *cheV* (11.76-fold change) and *cheR* (7.90-fold change) were also up-regulated substantially in expression when exposed to BZT.

**Figure 1:**
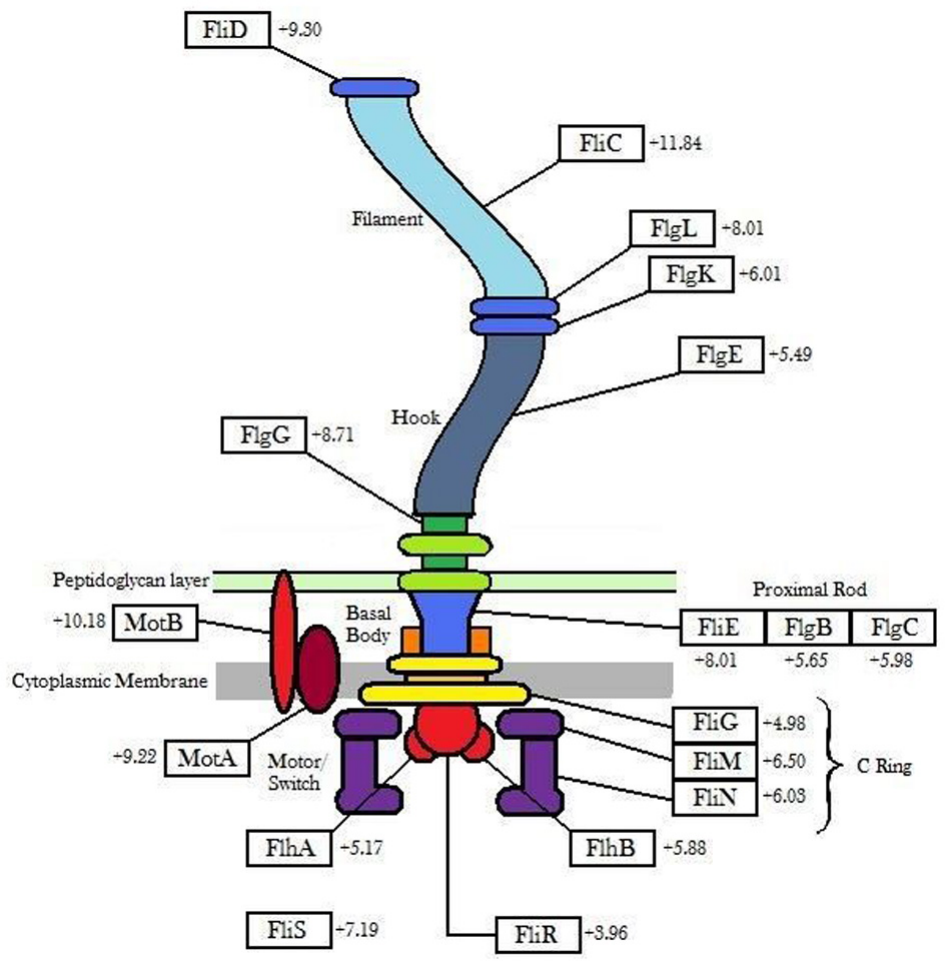
**Outline of flagellar genes (showing the degree of up-regulation) up-regulated in *Listeria monocytogenes* strain 6179 in the presence of BZT**.

Genes involved in flagellar biosynthesis also showed increased levels of expression compared to the controls (Figure 1). In particular, the *flaA* gene which codes for flagellin, the principal component of bacterial flagellum, was up-regulated by 11.84-fold in gene expression when 6179 was grown in the presence of the detergent. In addition to this, an up-regulation in the presence of BZT was also observed for the genes *motA* and *motB*. The MotA and MotB proteins, which together form the stator component of the flagellum itself, are integral membrane proteins that interact with the *fliG*, *fliM* and *fliN* genes, and serve a function in transmembrane transport of protons in the aforementioned flagellar motor complex (Tang et al., 1996). This transport of protons provides the energy required for the flagella to rotate, thus enabling the bacterial cell to become motile.

The three gene components that make up the flagellar motor switch complex, namely *fliG*, *fliM* and *fliN*, also exhibited an up-regulation in expression of 4.98-fold, 6.50-fold and 6.03-fold respectively. This complex (induced through interaction with the *cheY* gene), alters the rotation of the flagella from the default clockwise, to rotation in a counter clockwise direction. Rotation of the flagella in a clockwise direction allows the bacterial cell to experience “tumbling,” whereby the cell remains in a stationary position, simply rotating in place. However, once the rotation of the flagella is altered to the counter clockwise direction, the flagella will then align at one end of the cell to form a single rotating bundle, resulting in a smooth swimming behavior, and allowing the bacteria to physically move in a desired direction (Dons et al., 2004). In this way, once the presence of detergent is detected by the organism, up-regulation of these genes will allow the bacterial cell to become motile, and move away from the unfavorable environment in which it finds itself. Other genes up-regulated in the presence of BZT (some of which, as with other subsequent tables, fall below the 4-fold change in relative expression, yet remain biologically relevant to the results) include *fliP*, *fliQ*, *fliR* and *fliS*, as well as a number of specific flagellar structural genes such as *fliD* (encoding the flagellar capping protein), *flgK* (encoding a hook-associated protein), *flgC* (encoding a basal body rod protein), *flgE* (encoding a flagellar hook protein), *flgG* and *flgL* (Table 2).

**Table 2:** **Chemotaxis and flagellar assembly genes which change in regulation following exposure of strain 6179 to BZT**.

**Functional category**	**Locus tag**	**Gene**	**Protein**	**Fold change**
**UPREGULATED**				
*Chemotaxis*	LM6179_2457	*MCP*	Methyl-accepting chemotaxis protein	6.24
	LM6179_1003	*cheA*	Chemotactic two-component sensor histidine kinase	3.90
	LM6179_1002	*cheY*	Regulator of chemotaxis and motility	6.77
	LM6179_1000	*cheV*	Chemotaxis protein CheV	11.76
	LM6179_0994	*cheR*	Chemotaxis protein methyltransferase	7.90
*Flagellar assembly*	LM6179_1025	*fliG*	Putative flagellar switch protein	4.98
	LM6179_1010	*fliM*	Flagellar motor switch protein FliM	6.50
	LM6179_1011	*fliN*	Flagellar motor switch protein FliN	6.03
	LM6179_1001	*flaA*	Flagellin (aka FliC)	11.84
	LM6179_0996	*motA*	Motility protein A, MotA component of the stator flagellum complex	9.22
	LM6179_0997	*motB*	Motility protein B, MotB component of the stator flagellum complex	10.18
	LM6179_1018	*fliD*	Flagellar capping protein (FliD)	9.30
	LM6179_1016	*flgK*	Flagellar hook-associated protein FlgK	6.01
	LM6179_1022	*flgC*	Flagellar component of cell-proximal portion of basal-body rod	5.98
	LM6179_1008	*flgE*	Putative flagellar hook protein FlgE	5.49
	LM6179_0993	*flgG*	Flagellar basal body rod protein FlgG	8.71
	LM6179_1017	*flgL*	Flagellar hook-associated protein FlgL	8.01
	LM6179_1019	*fliS*	Flagellar biosynthesis protein fliS	7.19
	LM6179_0991	*flhA*	Flagellar biosynthesis protein FlhA	5.17
	LM6179_0990	*flhB*	Flagellar biosynthesis protein FlhB	5.88
	LM6179_0989	*fliR*	Flagellar biosynthesis protein FliR	3.96

A gene is considered to be up-regulated if its expression level is greater when the bacterium is grown in the presence of BZT than in the absence of BZT.

Such a considerable up-regulation of genes associated with chemotaxis and motility had not been reported in the previous study (Fox et al., 2011a,b). This is possibly due to a combination of the reference strain used, the sensitivity of the software, and indeed the high threshold set by Fox et al. given the fact that the bacterium's own host genome was unavailable at the time. However, the response itself is somewhat logical from bacteria facing such an unfavorable environment. In a similar fashion to how bacteria will interpret and utilize extracellular signals in order to move toward a nutrient source, they will also use these signals in order to elicit a chemotactic response and allow the cell to move away from a repellent (Porter et al., 2011). The noteworthy up-regulation of the MCP gene compared to controls may suggest that the cell is in a state of constant interaction with its surroundings, reassessing the extracellular conditions as it progresses, ultimately searching for an environment where the concentration of detergent is at a more tolerable level to allow the bacterium to survive. The chemotaxis and flagellar genes work in tandem with one another in order to allow bacteria to physically respond quickly and effectively to the environmental conditions surrounding them (Hazelbauer et al., 2008).

### Fatty acid biosynthesis/metabolism

This study also identified a considerable change in relative expression of a number of genes which are involved in fatty acid biosynthesis and metabolism in *L. monocytogenes*. In the presence of BZT, the *acpP* gene, which codes for the acyl carrier protein (ACP), was up-regulated by 18.25-fold relative to expression levels observed when the bacteria were grown in the absence of the detergent. *acpP* belongs to a cluster of genes which are involved in the biosynthesis of fatty acids and phospholipids, a cluster which also includes the genes *plsX*, *fabD*, *fabG*, *fabHA*, and *fabF* (Martinez et al., 2010). Like *acpP*, *plsX*, *fabD*, and *fabG* all demonstrate up-regulation in the presence of BZT (Table 3). The *fabHA* and *fabF* genes were also up-regulated; these two genes comprise the *fabHAF* operon, which encodes both FabH; a protein responsible for the initiation of fatty acid elongation, and FabF, which catalyzes the remaining elongation steps in this pathway (Schujman et al., 2003).

**Table 3:** **Fatty acid biosynthesis/metabolism genes which change in regulation following exposure of strain 6179 to BZT**.

**Functional category**	**Locus tag**	**Gene**	**Protein**	**Fold change**
**UPREGULATED**				
*Fatty acid Biosynthesis/Metabolism*	LM6179_2576	*acpP*	Acyl carrier protein	18.25
	LM6179_2579	*plsX*	Phosphate:acyl-ACP acyltransferase	4.37
	LM6179_2578	*fabD*	Malonyl CoA:acyl carrier protein transacylase	3.56
	LM6179_2577	*fabG*	Beta-ketoacyl-acyl carrier protein reductase	7.04
	LM6179_2981	*fabHA*	Beta-ketoacyl-acyl carrier protein synthase III	3.51
	LM6179_2980	*fabF*	Beta-ketoacyl-acyl carrier protein synthase II	3.78
	LM6179_1286	*fabI*	Enoyl-acyl carrier protein reductase	4.41
	LM6179_1679	*fabZ*	(3R)-hydroxymyristoyl-(acyl carrier protein) dehydratase	3.43
	LM6179_2444	*fabL*	Enoyl-acyl carrier protein reductase III	3.03
	LM6179_2323	*accA*	Acetyl-CoA carboxylase (carboxyltransferase alpha subunit)	2.37
	LM6179_2099	*accB*	Acetyl-CoA carboxylase subunit (biotin carboxyl carrier subunit)	1.79
	LM6179_2100	*accC*	Acetyl-CoA carboxylase subunit (biotin carboxylase subunit)	3.52
	LM6179_2324	*accD*	Acetyl-CoA carboxylase (carboxyltransferase beta subunit)	2.02
	LM6179_2580	*fapR*	Transcription factor controlling fatty acid and phospholipid metabolism	3.89

A gene is considered to be up-regulated if its expression level is greater when the bacterium is grown in the presence of BZT than in the absence of BZT.

Numerous other genes involved in fatty acid biosynthesis; *fabI* (4.41-fold), *fabZ* (3.43-fold), and *fabL* (3.03-fold), also exhibited an up-regulation in their expression under these conditions. In addition, genes *accA*, *accB*, *accC* and *accD*, which together encode the four subunits of the acetyl-CoA carboxylase enzyme, were also up-regulated, by 2.37-fold, 1.79-fold, 3.52-fold, and 2.02-fold respectively. The acetyl-CoA carboxylase enzyme is responsible for catalyzing the carboxylation of acetyl-CoA to produce malonyl-CoA, and is widely considered to be the major rate-limiting enzyme in fatty acid biosynthesis (Brownsey and Denton, 1987). The action of quaternary ammonium compounds (QAC) such as benzethonium chloride on the cell membrane of Gram-positive bacteria results in the solubilization of hydrophobic components in the membrane, such as phospholipids and lipoteichoic acids. This in turn causes a phase transition of the bacterial cell membrane from a fluid to a liquid crystalline state, and a subsequent loss of vital physiological and osmoregulatory functions of the cell itself, eventually resulting in cell death in high QAC concentrations (Gilbert and Moore, 2005). Therefore, the observation of up-regulation of genes involved in phospholipid biosynthesis when strain 6179 is exposed to BZT is somewhat expected, considering the effect that the detergent has on the fluidity of cell membrane. Previous studies (Najjar et al., 2007) have described how the membrane fluidity of *L. monocytogenes* can change in response to various stresses, such as that of a lowered growth temperature, and the maintenance of membrane fluidity under these stress conditions depends on the phospholipid composition within the membrane. In line with this, another gene which is indirectly involved in maintaining a fluid state of the cell membrane, namely *fapR*, was up-regulated in its expression in the presence of BZT by a factor of almost 4-fold. This gene encodes the transcription factor FapR, which has a pivotal role in the regulation of membrane phospholipid biosynthesis by repressing the expression of a number of genes that are involved in phospholipid and fatty acid metabolism (Schujman et al., 2003).

### Cobalamin biosynthesis

Another biosynthetic pathway that was up-regulated on exposure to BZT functions in the synthesis of cobalamin (vitamin B-12) from uroporphyrinogen III. In many Gram-positive bacteria, synthesis of cobalamin can be achieved through either an aerobic or anaerobic pathway. A different gene set is required for each of the pathways; the *cob* genes are required for the aerobic pathway, while the *cbi* genes are required for the anaerobic pathway (Raux et al., 1998). Upon exposure of the bacteria to BZT, several of the *cbi* genes considerably increased in their relative expression, by a range of 6-fold to 12.5-fold compared to controls, including the *cbiK, cbiL*, and *cbiH* genes amongst many others (Table 4)*.* In addition, there was also an observed increase in expression of genes up- and down-stream of the *cbi* genes in this pathway, including *cobA* (11.09-fold change), *cobD* (9.28-fold change), and *cobQ* (6.25-fold change), all of which function in both the aerobic and anaerobic pathways of cobalamin biosynthesis. Although the precise reason for up-regulation of cobalamin biosynthesis is unknown, it is well documented that many bacteria, including *Listeria monocytogenes* (Buchrieser et al., 2003), possess genes responsible for biosynthesis of cobalamin and indeed for use of cobalamin or other closely-related intermediates of the cobalamin biosynthetic pathway as cofactors for various different biological processes, including in the degradation of ethanolamine and 1,2-propanediol as a source of carbon and energy, and likewise in the synthesis of acetyl-CoA (Roth et al., 1996). In synthesizing acetyl-CoA, this pathway may also serve a function in feeding into the aforementioned fatty acid biosynthesis pathway, suggesting the up-regulation of these particular genes contributes to the maintenance of a functioning membrane under severe environmental stress.

**Table 4:** **Cobalamin biosynthesis genes which change in regulation following exposure of strain 6179 to BZT**.

**Functional category**	**Locus tag**	**Gene**	**Protein**	**Fold change**
**UPREGULATED**				
*Cobalamin biosynthesis*	LM6179_1509	*cbiK*	Sirohydrochlorin cobaltochelatase	15.24
	LM6179_1510	*cbiL*	Precorrin-2 C20-methyltransferase	12.46
	LM6179_1506	*cbiH*	Cobalt-precorrin-3B C(17)-methyltransferase	8.73
	LM6179_1504	*cbiF*	Cobalt-precorrin-4 C(11)-methyltransferase	5.97
	LM6179_1505	*cbiG*	CbiG protein	7.05
	LM6179_1501	*cbiD*	Putative cobalt-precorrin-6A synthase (deacetylating)	8.40
	LM6179_1507	*cbiJ/cobK*	Precorrin-6X reductase	9.35
	LM6179_1502	*cbiE*	Putative cobalt-precorrin-6Y C(5)-methyltransferase	10.05
	LM6179_1503	*cbiT*	Putative cobalt-precorrin-6Y C(15)-methyltransferase (decarboxylating)	10.27
	LM6179_1500	*cbiC*	Cobalt-precorrin-8X methylmutase	8.66
	LM6179_1498	*cobB/cbiA*	Cobyrinic acid A,C-diamide synthase	6.61
	LM6179_1508	*cobA*	Uroporphyrin-III C-methyltransferase/uroporphyrinogen-III synthase	11.09
	LM6179_1499	*cobD*	Cobalamin biosynthesis protein CobD	9.28
	LM6179_1515	*cobQ*	Cobyric acid synthase	6.25

A gene is considered to be up-regulated if its expression level is greater when the bacterium is grown in the presence of BZT than in the absence of BZT.

### Peptidoglycan biosynthesis

One of the most noteworthy changes observed in this study was the widespread up-regulation in the expression of genes and pathways which ultimately have a role in the biosynthesis of peptidoglycan (PG), the principal component of the Gram-positive bacterial cell wall. These include the *mur* family of PG biosynthesis genes (El Zoeiby et al., 2003), namely *murC* (1.96-fold), *murD* (4.44-fold), *murE* (4.48-fold) and *murF* (3.89-fold), which encode enzymes that function together with the *pdp* (2.32-fold) and *yodJ* (3.16-fold) genes in the conversion UDP-N-Acetylmuramate to peptidoglycan. Also found up-regulated in the presence of BZT are genes involved in the synthesis of the UDP-N-Acetylmuramate precursor itself from fructose-6-phosphate, including *gtaB* (5.55-fold), *glmM* (4.29-fold), *glmS* (3.36-fold), and *gcaD* (3.07-fold). The up-regulation of PG biosynthesis genes is somewhat expected considering the action of QAC's such as BZT involves disruption of the bacterial cell wall, and similar observations to these have previously been shown in the study by Fox et al. (2011a,b).

Indirectly associated with this PG biosynthesis pathway are a number of other genes which are involved in conferring structural reinforcement to the outer cell wall of *L. monocytogenes*. Included within this category are genes which code for proteins whose main function are in the synthesis of teichoic acids and polysaccharides. For example, the *mnaA* gene, which has been previously identified (Dubail et al., 2006) to play an essential role in teichoic acid biogenesis in *L. monocytogenes*, was up-regulated 3.64-fold in the presence of BZT. Additionally, the *tagG* and *tagH* genes, which together form the *tagGH* operon, were also up-regulated in the presence of the detergent, by folds of 7.11 and 10.91 respectively. This operon is homologous to that of the *tagGH* operon in *Bacillus subtilis* strain 168, which functions in translocation of teichoic acids through the cytoplasmic membrane (Lazarevic and Karamata, 1995). In a similar fashion, numerous other genes which also play a role in teichoic acid biosynthesis and transport are also found to increase in their relative expression under these conditions (Table 5), including *ggaB* (6.45-fold change), *tagD* (5.55-fold change), *gtcA* (5.42-fold change), and *tagO* (3.06-fold change).

**Table 5:** **Peptidoglycan and teichoic acid biosynthesis genes which change in regulation following exposure of strain 6179 to BZT**.

**Functional category**	**Locus tag**	**Gene**	**Protein**	**Fold change**
**UPREGULATED**				
*Peptidoglycan biosynthesis*	LM6179_1014	*murB*	UDP-N-acetylenolpyruvoylglucosamine reductase	7.80
	LM6179_2355	*murC*	UDP-N-acetyl muramate-alanine ligase	1.97
	LM6179_2808	*murD*	UDP-N-acetylmuramoylalanyl-D-glutamate ligase	4.45
	LM6179_2810	*murE*	UDP-N-acetylmuramoylalanyl-D-glutamate-2, 6-diaminopimelate ligase	4.49
	LM6179_1168	*murF*	UDP-N-acetylmuramoylalanyl-D-glutamyl-2, 6-diaminopimelate-D-alanyl-D-alanine ligase	3.89
	LM6179_2811	*pbpB*	Penicillin-binding protein 2B	2.33
	LM6179_2625	*yodJ*	D-alanyl-D-alanine carboxypeptidase lipoprotein	3.17
	LM6179_1398	*gtaB*	UTP-glucose-1-phosphate uridylyltransferase	5.55
	LM6179_2893	*glmM*	Phosphoglucosamine mutase	4.29
	LM6179_1039	*glmS*	L-glutamine-D-fructose-6-phosphate amidotransferase (AKA Glucosamine–fructose-6-phosphate aminotransferase)	3.36
	LM6179_0488	*gcaD*	Bifunctional glucosamine-1-phosphate N-acetyltransferase/UDP-N-acetylglucosamine pyrophosphorylase	3.07
*Teichoic acid biosynthesis*	LM6179_1666	*mnaA*	UDP-N-acetylmannosamine 2-epimerase	3.64
	LM6179_1395	*tagH*	Teichoic acids export ATP-binding protein TagH	10.91
	LM6179_1394	*tagG*	Teichoic acid precursors permease	7.11
	LM6179_1400	*ggaB*	Minor teichoic acids biosynthesis protein GgaB	6.45
	LM6179_1409	*tagD*	Glycerol-3-phosphate cytidylyltransferase	5.55
	LM6179_1653	*gtcA*	Cell wall teichoic acid glycosylation protein gtcA	5.42
	LM6179_1684	*tagO*	UDP-N-acetylglucosamine:undecaprenyl-P N-acetylglucosaminyl-1-P transferase	3.06
	LM6179_1401	*rfbA*	Glucose-1-phosphate thymidylyltransferase	7.25
	LM6179_1403	*rfbB*	dTDP-glucose 4,6-dehydratase	6.46
	LM6179_1402	*rfbC*	dTDP-4-deoxyrhamnose-3,5-epimerase	8.85
	LM6179_1404	*rfbD*	dTDP-4-dehydrorhamnose reductase	6.93

A gene is considered to be up-regulated if its expression level is greater when the bacterium is grown in the presence of BZT than in the absence of BZT.

Equally, a group of four genes, which together comprise the *rfbABCD* operon, were also up-regulated relative to controls upon exposure of strain 6179 to BZT. These genes, namely *rfbA*, *rfbB*, *rfbC* and *rfbD*, exhibited an increase in relative expression by 7.25-fold, 6.46-fold, 8.85-fold, and 6.93-fold respectively, under stress. The *rfbABCD* operon is involved in the biosynthesis of dTDP-L-Rhamnose, which is an important component of cell wall polysaccharides in many Gram-positive bacteria (Boels et al., 2004).

### PTS system

Following exposure of the bacterium to BZT, several genetic components of the phosphotransferase system (PTS) were found to be up-regulated compared to controls. The *ptsH* and *ptsI* genes, which together form the *ptsHI* operon, are up-regulated in their expression by 4.33-fold and 4.47-fold respectively, when strain 6179 is grown in the presence of BZT. The *ptsHI* operon encodes two components of the phosphoenolpyruvate (PEP)-dependent PTS, namely HPr (*ptsH*) and Enzyme I (*ptsI*), which function in glucose uptake into the bacterial cell (Christensen et al., 1998). In addition, the *ptsG* gene, which encodes a major glucose-specific transporter in *L. monocytogenes* (Stoll and Goebel, 2010) is up-regulated 11.72-fold in the presence of BZT. The *bglPH* operon, consisting of the genes *bglP* (27.79-fold change) and *bglH* (11.11-fold change) was also found to be up-regulated in the presence of the detergent. This operon encodes two further enzymatic components of the PEP-dependent PTS in Gram-positive bacteria (Krüger and Hecker, 1995).

Numerous other genes encoding subunits or components of the phosphotransferase system also exhibited up-regulation following exposure to BZT of a range between 20- to 70-fold in expression compared to controls (Table 6). The PTS system is responsible for the phosphorylation and translocation of sugars across the cell membrane. Upon exposure to the QAC, the bacterium responds by up-regulating a number of pathways, such as those of peptidoglycan biosynthesis, which require carbohydrates, or indeed products of carbohydrate metabolism as precursors. When strain 6179 is grown in BZT, components of the PTS-system which are specifically involved in the uptake of glucose, fructose, and mannose are up-regulated in their relative expression. Glucose and fructose are fed into the glycolysis pathway in order to be converted to fructose-6-phosphate, which ultimately serves as a specific precursor for the PG biosynthesis pathway. Consequently, an increase in expression of a number of genes involved in the glycolysis pathway is also observed upon exposure of strain 6179 to the detergent, including a 29.83-fold up-regulation of the phosphoglyceromutase gene *gpmA*, a 5.33-fold up-regulation of phosphoglucomutase gene *pgcA*, and an up-regulation of a number of genes coding for various kinases including glucose kinase *glcK* (4.21-fold), pyruvate kinase *pyk* (3.31-fold), and acetate kinase *ackA* (2.74-fold).

**Table 6:** **Phosphotransferase system and glycolysis genes which change in regulation following exposure of strain 6179 to BZT**.

**Functional category**	**Locus tag**	**Gene**	**Protein**	**Fold change**
**UPREGULATED**				
*Phosphotransferase system*	LM6179_1320	*ptsI*	Phosphotransferase system (PTS) enzyme I	4.47
	LM6179_1319	*ptsH*	Histidine-containing phosphocarrier protein of the phosphotransferase system (PTS) (HPr protein)	4.33
	LM6179_1334	*ptsG*	Putative PTS system, glucose-specific, IIA component	11.72
	LM6179_0306	*bglP*	PTS system beta-glucoside-specific EIIBCA component	27.79
	LM6179_0618	*bglH*	aryl-phospho-beta-d-glucosidase	11.11
	LM6179_2766	LM6179_2766	PTS system protein	69.14
	LM6179_2769	LM6179_2769	PTS system mannose/fructose/sorbose family IID subunit	66.88
	LM6179_2770	LM6179_2770	PTS system protein	48.23
	LM6179_2771	LM6179_2771	PTS system mannose/fructose/sorbose family IIB subunit	21.06
*Glycolysis*	LM6179_0815	*gpmA*	Phosphoglycerate mutase family protein	29.83
	LM6179_2641	*pgcA*	Phosphoglucomutase/phosphomannomutase	5.33
	LM6179_2081	*glcK*	Glucose kinase	4.21
	LM6179_2321	*pyk*	Pyruvate kinase	3.31
	LM6179_2332	*ackA*	Acetate kinase	2.74

A gene is considered to be up-regulated if its expression level is greater when the bacterium is grown in the presence of BZT than in the absence of BZT.

### Stress response

As expected when subjected to a harsh environment, *L. monocytogenes* increases expression levels of several stress response genes. Included in these are *cspC* (9.25-fold) and *cspD* (4.84-fold) genes, both encoding cold shock proteins. Similarly, there is a notable increase in expression of a number of heat shock genes, including *rsbRA* (4.22-fold), *ftsH* (3.70-fold), *hslR* (3.53-fold), and *hslO* (3.40-fold), among other general stress genes such as *yloU* (4.76-fold) which encodes an alkaline shock protein, and *liaR* (3.68-fold), which encodes a regulatory protein that responds to cell stress. While some of the observations made in terms of stress response may be attributed to cross-protection, the increased expression of a number of these genes is indicative of the bacteria's attempt to adapt to the harsh new environment in which it finds itself.

### Multi-drug resistance transporters

A considerable increase in expression of numerous genes encoding multi-drug resistance transporters is also noted when this strain of *L. monocytogenes* is exposed to BZT. Inclusive in these is a marked 4-fold increase in expression of the quaternary ammonium compound resistance protein, *qacH*, which has previously been identified (Muller et al., 2013) to have a pivotal role in increased resistance of *L. monocytogenes* to QAC exposure. In addition, there was a 3-fold increase in expression of two genes, *ykkC* and *lmrB*, which encode efflux transporters, as well as an increase in expression of several other genes which are also involved in multi-drug resistance (Table 7).

**Table 7:** **Stress response, multi-drug resistance transporter, and phage/prophage genes which change in regulation following exposure of strain 6179 to BZT**.

**Functional category**	**Locus tag**	**Gene**	**Protein**	**Fold change**
**UPREGULATED**				
*Stress response*	LM6179_2107	*cspC*	Cold-shock protein	9.25
	LM6179_2786	*cspD*	Cold-shock protein, molecular chaperone, RNA-helicase co-factor	4.84
	LM6179_1203	*rsbRA*	Component of the piezosome (stressosome), positive regulation of sigma(B) activity in response to salt and heat stress	4.22
	LM6179_0510	*ftsH*	Cell-division protein and general stress protein (class III heat-shock)	3.70
	LM6179_0506	*hslR*	Ribosomal RNA binding protein involved in 50S recycling, heat shock protein	3.53
	LM6179_0512	*hslO*	Disulfide bond chaperone (heat shock protein HSP33)	3.40
	LM6179_2585	*yloU*	Putative alkaline-shock protein	4.76
*Multi-drug resistance*	LM6179_2296	*qacH*	Quaternary ammonium compound-resistance protein	4.01
*Transporters*	LM6179_1165	*ykkC*	Efflux transporter	3.71
	LM6179_0824	*lmrB*	Efflux transporter, drug-export protein	3.25
	LM6179_1166	*ykkD*	Efflux transporter	2.75
	LM6179_2153	LM6179_2153	Multidrug resistance transporter	2.23
	LM6179_1610	*ycnB*	Putative efflux transporter	2.08
	LM6179_1298	LM6179_1298	Drug resistance transporter, EmrB/QacA family	1.76
	LM6179_1894	*yuxJ*	Putative exporter	1.69
	LM6179_0246	LM6179_0246	Efflux protein	1.50
	LM6179_0155	*mdtG*	Putative metabolite efflux transporter	1.27
	LM6179_2368	*ycnB*	Uncharacterized MFS-type transporter ycnB	1.20
**DOWNREGULATED**				
*Phage/prophage*	LM6179_1786	LM6179_1786	Bacteriophage major tail shaft protein	−11.35
	LM6179_1780	LM6179_1780	Phage—major head protein	−7.45
	LM6179_1597	LM6179_1597	Phage tail tape measure protein	−6.60
	LM6179_1588	LM6179_1588	Cps major phage capsid protein	−4.98
	LM6179_1968	LM6179_1968	Phage holin protein	−14.49
	LM6179_1777	LM6179_1777	Phage portal protein	−5.66
	LM6179_1580	LM6179_1580	Phage-related transcriptional activator	−3.74

A gene is considered to be up-regulated if its expression level is greater when the bacterium is grown in the presence of BZT than in the absence of BZT.

### Phage/prophage

In contrast to other genes, those encoding phage proteins were found to be considerably down-regulated when strain 6179 was exposed to the QAC. In total, over 75 genes which fall into this category were found to decrease in expression by at least 3-fold compared to the levels seen in the control experiment. These genes include uncharacterized phage proteins, as well as structural phage proteins, such as the major tail shift protein (11.35-fold change), major head protein (7.45-fold change), tail tape measure protein (6.60-fold change) and the major capsid protein (4.98-fold change). In addition to these, functional phage proteins such as the phage holin protein (14.49-fold change), phage portal protein (5.66-fold change) and a phage-related transcriptional activator protein (3.74-fold change) were also found to be down-regulated in the presence of BZT. Although the observed down-regulation in phage gene expression is quite substantial when strain 6179 is exposed to the detergent, the reason as to why such a change occurs is still as yet relatively unknown, and further experimentation will be required in order to fully understand the underlying mechanisms at work. A study on the response of *Staphylococcus aureus* to a mild acid (Weinrick et al., 2004) revealed a similar effect on prophage gene expression to that seen here, suggesting that the drop in pH from 7.5 to 5.5 resulted in a less frequent spontaneous induction of prophage. Considering that the pH of a 1% aqueous solution of BZT is somewhere in the range of 4.8–5.5, the observed down-regulation of prophage genes in the test sample could potentially be attributed to the mildly acidic environment compared to the control.

### Concluding remarks

The results obtained from the transcriptome sequence analysis suggest that the response of *L. monocytogenes* to the presence of BZT is essentially two-fold; an up-regulation in genes and gene pathways which are directly or indirectly involved in cell wall biosynthesis, and equally an up-regulation in genes that code for proteins and other components which comprise the organism's chemotaxis and motility cascade. In addition to these two main responses, the bacterium also up-regulates many genes involved in maintenance of cell membrane fluidity, as well as those involved in uptake of carbohydrates, in order to adapt to the presence of BZT. Expression of genes encoding virulence factors, such as listeriolysin O and the internalins, remained relatively unchanged following exposure of the bacteria to BZT (data not shown). In the study by Fox et al. (2011a,b), sequenced mRNA reads from this particular strain of the bacteria were mapped and quantified against a similar reference genome, namely *L. monocytogenes* strain f6854. While the results of that study made some similar observations to those discussed here where peptidoglycan biosynthesis and the phosphotransferase system are concerned, the considerable up-regulation in chemotaxis and motility genes is an observation only reported in this particular study. As previously mentioned, the primary difference between the two studies concerned the reference strain used in read mapping, in that the host's own fully sequenced genome was available for this research. While using a highly similar genome as a reference for this type of work does give us some insight into the response of *L. monocytogenes* to such an environmental stress as exposure to BZT, it is clear from the findings that in order to fully ascertain the bacterial response, it is more favorable to use the bacterium's own genome where possible.

From a persistence perspective, it is now generally thought that a bacterium's ability to persist in a given environment can be attributed to a combination of factors, rather than a single individual trait. This study investigated the response of a persistent strain of *L. monocytogenes* when exposed to a sub-lethal concentration of an industrial detergent, and, while providing an insight on a genetic level as to how this strain is able to persist and adapt to the introduction of such a threat to its survival, the results are not a complete representation of the phenomenon of persistence itself. Exposure of the bacterium to such a low level of detergent in the food processing environment would only occur as a result of improper cleaning practices, considering the extremely high concentrations at which they are generally applied to equipment and surfaces. The ability of the bacterium to form a biofilm, as well as its colonization of niche areas, such as corners of machinery or other hard to reach places, can result in the bacterium being exposed to these substantially reduced detergent concentrations, and these factors may also contribute in some part to the ability of the bacterium to achieve persistence.

### Conflict of interest statement

The authors declare that the research was conducted in the absence of any commercial or financial relationships that could be construed as a potential conflict of interest.

## References

[B1] Boels I. C. Beerthuyzen M. M. Kosters M. H. W. Van Kaauwen M. P. W. Kleerebezem M. de Vos W. M. (2004). Identification and functional characterization of the Lactococcus lactis rfb operon, required for dTDP-rhamnose biosynthesis. J. Bacteriol. 186, 1239–1248 10.1128/JB.186.5.1239-1248.2004 14973085 PMC344400

[B2] Brownsey R. W. Denton R. (1987). Acetyl-coenzyme A carboxylase. Enzymes 18, 123–146 10.1016/S1874-6047(08)60256-5

[B3] Buchrieser C. Rusniok C. Kunst F. Cossart P. Glaser P. (2003). Comparison of the genome sequences of *Listeria monocytogenes* and Listeria innocua: clues for evolution and pathogenicity. FEMS Immunol. Med. Microbiol. 35, 207–213 10.1016/S0928-8244(02)00448-0 12648839

[B4] Christensen D. P. Benson A. K. Hutkins R. W. (1998). Cloning and expression of the *Listeria monocytogenes* scott A ptsH and ptsI genes, coding for HPr and enzyme I, respectively, of the phosphotransferase system. Appl. Environ. Microbiol. 64, 3147–3152 9726852 10.1128/aem.64.9.3147-3152.1998 PMC106702

[B5] Derr P. Boder E. Goulian M. (2006). Changing the specificity of a bacterial chemoreceptor. J. Mol. Biol. 355, 923–932 10.1016/j.jmb.2005.11.025 16359703

[B6] Dons L. Eriksson E. Jin Y. Rottenberg M. E. Kristensson K. Larsen C. N. (2004). Role of flagellin and the two-component CheA/CheY system of *listeria monocytogenes* in host cell invasion and virulence. Infect. Immun. 72, 3237–3244 10.1128/IAI.72.6.3237-3244.2004 15155625 PMC415653

[B7] Dubail I. Bigot A. Lazarevic V. Soldo B. Euphrasie D. Dupuis M. (2006). Identification of an essential gene of *listeria monocytogenes* involved in teichoic acid biogenesis. J. Bacteriol. 188, 6580–6591 10.1128/JB.00771-06 16952950 PMC1595501

[B8] El Zoeiby A. Sanschagrin F. Levesque R. C. (2003). Structure and function of the Mur enzymes: development of novel inhibitors. Mol. Microbiol. 47, 1–12 10.1046/j.1365-2958.2003.03289.x 12492849

[B9] Fox E. Hunt K. O'Brien M. Jordan K. (2011a). *Listeria monocytogenes* in Irish Farmhouse cheese processing environments. Int. J. Food Microbiol. 145(Suppl. 1), S39–S45 10.1016/j.ijfoodmicro.2010.10.012 21087802

[B10] Fox E. M. Leonard N. Jordan K. (2011b). Physiological and transcriptional characterization of persistent and nonpersistent *listeria monocytogenes* isolates. Appl. Environ. Microbiol. 77, 6559–6569 10.1128/AEM.05529-11 21764947 PMC3187160

[B11] Gandhi M. Chikindas M. L. (2007). Listeria: a foodborne pathogen that knows how to survive. Int. J. Food Microbiol. 113, 1–15 10.1016/j.ijfoodmicro.2006.07.008 17010463

[B12] Gilbert P. Moore L. E. (2005). Cationic antiseptics: diversity of action under a common epithet. J. Appl. Microbiol. 99, 703–715 10.1111/j.1365-2672.2005.02664.x 16162221

[B13] Guenther S. Huwyler D. Richard S. Loessner M. J. (2009). Virulent bacteriophage for efficient biocontrol of *listeria monocytogenes* in ready-to-eat foods. Appl. Environ. Microbiol. 75, 93–100 10.1128/AEM.01711-08 19011076 PMC2612219

[B14] Hazelbauer G. L. Falke J. J. Parkinson J. S. (2008). Bacterial chemoreceptors: high-performance signaling in networked arrays. Trends Biochem. Sci. 33, 9–19 10.1016/j.tibs.2007.09.014 18165013 PMC2890293

[B15] Holck A. Berg J. (2009). Inhibition of *Listeria monocytogenes* in cooked ham by virulent bacteriophages and protective cultures. Appl. Environ. Microbiol. 75, 6944–6946 10.1128/AEM.00926-09 19717623 PMC2772455

[B16] Kastbjerg V. G. Gram L. (2009). Model systems allowing quantification of sensitivity to disinfectants and comparison of disinfectant susceptibility of persistent and presumed nonpersistent *Listeria monocytogenes*. J. Appl. Microbiol. 106, 1667–1681 10.1111/j.1365-2672.2008.04134.x 19226386

[B17] Kathariou S. (2002). *Listeria monocytogenes* virulence and pathogenicity, a food safety perspective. J. Food Prot. 65, 1811–1829 12430709 10.4315/0362-028x-65.11.1811

[B18] Krüger S. Hecker M. (1995). Regulation of the putative bglPH operon for aryl-beta-glucoside utilization in Bacillus subtilis. J. Bacteriol. 177, 5590–5597 7559347 10.1128/jb.177.19.5590-5597.1995 PMC177369

[B19] Laksanalamai P. Joseph L. A. Silk B. J. Burall L. S. Tarr C. L. Gerner-Smidt P. (2012). Genomic characterization of *Listeria monocytogenes* strains involved in a multistate listeriosis outbreak associated with cantaloupe in US. PLoS ONE 7:e42448 10.1371/journal.pone.0042448 22860127 PMC3409164

[B20] Lazarevic V. Karamata D. (1995). The tagGH operon of Bacillus subtilis 168 encodes a two-component ABC transporter involved in the metabolism of two wall teichoic acids. Mol. Microbiol. 16, 345–355 10.1111/j.1365-2958.1995.tb02306.x 7565096

[B21] Lundén J. Autio T. Markkula A. Hellström S. Korkeala H. (2003). Adaptive and cross-adaptive responses of persistent and non-persistent *Listeria monocytogenes* strains to disinfectants. Int. J. Food Microbiol. 82, 265–272 10.1016/S0168-1605(02)00312-4 12593929

[B22] Martinez M. A. de Mendoza D. Schujman G. E. (2010). Transcriptional and functional characterization of the gene encoding acyl carrier protein in Bacillus subtilis. Microbiology 156(Pt 2), 484–495 10.1099/mic.0.033316-0 19850612

[B23] Meyer B. (2006). Does microbial resistance to biocides create a hazard to food hygiene? Int. J. Food Microbiol. 112, 275–279 10.1016/j.ijfoodmicro.2006.04.012 16769146

[B24] Mortazavi A. Williams B. A. McCue K. Schaeffer L. Wold B. (2008). Mapping and quantifying mammalian transcriptomes by RNA-Seq. Nat. Methods 5, 621–628 10.1038/nmeth.1226 18516045 PMC13303166

[B25] Muller A. Rychli K. Muhterem-Uyar M. Zaiser A. Stessl B. Guinane C. M. (2013). Tn6188—a novel transposon in *Listeria monocytogenes* responsible for tolerance to benzalkonium chloride. PLoS ONE 8:e76835 10.1371/journal.pone.0076835 24098567 PMC3788773

[B26] Najjar M. B. Chikindas M. Montville T. J. (2007). Changes in *Listeria monocytogenes* membrane fluidity in response to temperature stress. Appl. Environ. Microbiol. 73, 6429–6435 10.1128/AEM.00980-07 17704268 PMC2075051

[B27] Nelson K. E. Fouts D. E. Mongodin E. F. Ravel J. DeBoy R. T. Kolonay J. F. (2004). Whole genome comparisons of serotype 4b and 1/2a strains of the food−borne pathogen *Listeria monocytogenes* reveal new insights into the core genome components of this species. Nucleic Acids Res. 32, 2386–2395 10.1093/nar/gkh562 15115801 PMC419451

[B28] Porter S. L. Wadhams G. H. Armitage J. P. (2011). Signal processing in complex chemotaxis pathways. Nat. Rev. Microbiol. 9, 153–165 10.1038/nrmicro2505 21283116

[B29] Raux E. Lanois A. Warren M. J. Rambach A. Thermes C. (1998). Cobalamin (vitamin B12) biosynthesis: identification and characterization of a Bacillus megaterium cobI operon. Biochem. J. 335, 159–166 9742225 10.1042/bj3350159 PMC1219764

[B30] Roth J. Lawrence J. Bobik T. (1996). Cobalamin (coenzyme B12): synthesis and biological significance. Annu. Rev. Microbiol. 50, 137–181 10.1146/annurev.micro.50.1.137 8905078

[B31] Schujman G. E. Paoletti L. Grossman A. D. de Mendoza D. (2003). FapR, a bacterial transcription factor involved in global regulation of membrane lipid biosynthesis. Dev. Cell 4, 663–672 10.1016/S1534-5807(03)00123-0 12737802

[B32] Stoll R. Goebel W. (2010). The major PEP-phosphotransferase systems (PTSs) for glucose, mannose and cellobiose of *Listeria monocytogenes*, and their significance for extra- and intracellular growth. Microbiology 156, 1069–1083 10.1099/mic.0.034934-0 20056707

[B33] Tang H. Braun T. F. Blair D. F. (1996). Motility protein complexes in the bacterial flagellar motor. J. Mol. Biol. 261, 209–221 10.1006/jmbi.1996.0453 8757288

[B34] Vázquez-Boland J. A. Kuhn M. Berche P. Chakraborty T. Domínguez-Bernal G. Goebel W. (2001). Listeria pathogenesis and molecular virulence determinants. Clin. Microbiol. Rev. 14, 584–640 10.1128/CMR.14.3.584-640.2001 11432815 PMC88991

[B35] Weinrick B. Dunman P. M. McAleese F. Murphy E. Projan S. J. Fang Y. (2004). Effect of mild acid on gene expression in Staphylococcus aureus. J. Bacteriol. 186, 8407–8423 10.1128/JB.186.24.8407-8423.2004 15576791 PMC532443

